# Using the Pulse Contour Method to Measure the Changes in Stroke Volume during a Passive Leg Raising Test

**DOI:** 10.3390/s18103420

**Published:** 2018-10-12

**Authors:** Chun-Hung Su, Shing-Hong Liu, Tan-Hsu Tan, Chien-Hsien Lo

**Affiliations:** 1 Institute of Medicine, School of Medicine, Chung-Shan Medical University; Department of Internal Medicine, Chung-Shan Medical University Hospital, Taichung 402, Taiwan; such197408@gmail.com; 2 Department of Computer Science and Information Engineering, Chaoyang University of Technology, Taichung 413, Taiwan; 3 Department of Electrical Engineering, National Taipei University of Technology, Taipei 10608, Taiwan; thtan@ntut.edu.tw; 4 Department of Internal Medicine, Chung-Shan Medical University Hospital, Taichung 402, Taiwan; myintoolwin@gmail.com

**Keywords:** pulse contour method, stroke volume, flow sensor, blood pressure monitor

## Abstract

The pulse contour method is often used with the Windkessel model to measure stroke volume. We used a digital pressure and flow sensors to detect the parameters of the Windkessel model from the pulse waveform. The objective of this study was to assess the stability and accuracy of this method by making use of the passive leg raising test. We studied 24 healthy subjects (40 ± 9.3 years), and used the Medis^®^ CS 1000, an impedance cardiography, as the comparing reference. The pulse contour method measured the waveform of the brachial artery by using a cuff. The compliance and resistance of the peripheral artery was detected from the cuff characteristics and the blood pressure waveform. Then, according to the method proposed by Romano et al., the stroke volume could be measured. This method was implemented in our designed blood pressure monitor. A passive leg raising test, which could immediately change the preloading of the heart, was done to certify the performance of our method. The pulse contour method and impedance cardiography simultaneously measured the stroke volume. The measurement of the changes in stroke volume using the pulse contour method had a very high correlation with the Medis^®^ CS 1000 measurement, the correlation coefficient of the changed ratio and changed differences in stroke volume were *r*^2^ = 0.712 and *r*^2^ = 0.709, respectively. It was shown that the stroke volume measured by using the pulse contour method was not accurate enough. But, the changes in the stroke volume could be accurately measured with this pulse contour method. Changes in stroke volume are often used to understand the conditions of cardiac preloading in the clinical field. Moreover, the operation of the pulse contour method is easier than using impedance cardiography and echocardiography. Thus, this method is suitable to use in different healthcare fields.

## 1. Introduction

The hemodynamic characteristics of hearts have shown that high correlation with cardiovascular diseases, such as blood pressure (BP), cardiac output (CO), and systemic vascular resistance (SVR). Stroke volume (SV) and CO are fundamental measures of cardiovascular functions, and are essential for the accurate understanding of cardiovascular pathophysiology, the guidance of fluid, and vasoactive therapies [1]. The measurement of BP, CO, and SVR are important when managing critically ill patients. For example, patients who undergo high-risk surgical procedures need continuous post-surgery monitoring, and early hemodynamic optimization has shown to be very important, as it results in significantly reduced mortality [2,3].

BP and SV are generally measured using different medical instruments. The oscillometry and auscultation methods are often used to measure BP [4], and echocardiography [5] and impedance cardiography (ICG) [6] are noninvasive methods to measure SV. These instruments, used for the echocardiography and the ICG techniques, are all expensive and need an expert to process the results gained from them. Although less invasive and noninvasive measurement techniques are available, they do not provide precise enough results to satisfy clinical requirements. A recent meta-analysis reviewed by Joosten et al. found that none of these devices are able to provide accurate enough measurements in clinical settings [7]. Therefore, if it was possible to measure the changes in SV using a simple and easy technique, this technique would be the preferred method in health care.

The lifting of the legs is a rescue maneuver that has been used for years by first-aid rescuers. Passive leg raising (PLR) has recently gained interest as a test for monitoring functional hemodynamics and assessing fluid responsiveness since it is a simple way to transiently increase the cardiac preload [8]. When the legs are lifted, a part of the blood contained in the calves, approximately 150 mL, flows back to the right ventricle increasing right cardiac preloading. If the right ventricle is preload responsive, the increase in the systemic venous return results in an increase in the right cardiac output and hence in left ventricular filling [8]; the CO of the left ventricle also increases [9]. So, PLR could be considered a reversible volume challenge for the heart. In this regard, arterial pulse pressure and SV have been used to predict the response of cardiac preloading [10]. The Frank–Starling curves describe the relation between the cardiac preload and SV according to individual factors such as cardiac contractility, which could show fluid responsiveness in critically ill patients [8].

The pulse contour method, which measures CO using the Windkessel model, makes use of a nonlinear three-element model to compute the SV of each beat [11]. In the Windkessel model, the relation between the BP and CO is a first-order ordinary differential equation which proved that the total peripheral resistance and arterial compliance are coefficients. Liu et al. proposed a method to detect the SV using the pulse contour of the brachial artery which has been implemented in an oscillometric blood pressure monitor [12]. In their study, the SV measurement was compared with echocardiography. Their correlation coefficients were *r*^2^ = 0.462 and *r*^2^ = 0.498 for the male and female groups, respectively. These results were not accurate enough to ensure that this method will be used to measure SV.

PLR tests the preloading of the left ventricle and compares the difference in SV before and during PLR. We hypothesized that the changes in stroke volume could be estimated by the pulse contour using the brachial artery. The goal of this study was to measure the SV by using the pulse signal with the PLR test, and to analyze the limitations of this method. An instrument with the ICG technique, the Medis^®^ CS 1000, measured the SV, and we used that SV as the comparing reference in this study. We studied the accuracy of the SV and the changes in the SV, using the pulse contour method before and during the PLR test.

## 2. Methods

### 2.1. Pulse Contour Measurement

**Figure 1:**
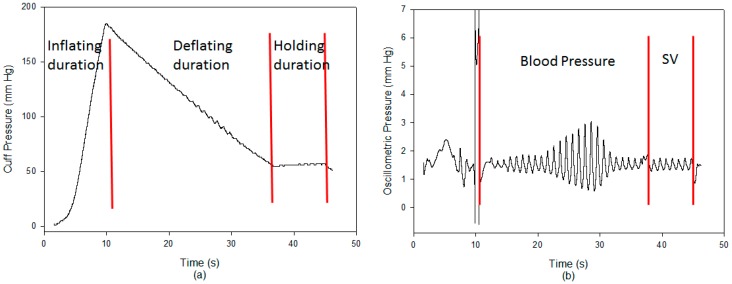
(**a**) The cuff pressure has three phases, inflating duration, deflating duration, and holding duration. (**b**) The oscillometric signal was used to measure the blood pressure, the pulse signal was used to measure the stroke volume during the holding duration.

The pulse contour method, proposed by Liu et al. [12], was implemented in our designed blood pressure monitor. Its microprocessor is STM32L476VC LQFP10, its digital pressure and flow sensors are FPS 520 and FDF 400 (Formosa Measurement Technology Inc. Ltd., Hsinchun, Taiwan). The bandwidths of the filters are 0.3 Hz to 4 Hz for the blood pressure measurement, and 0.3 Hz to 20 Hz for the stroke volume measurement. The sample rate used was 125 Hz. The signals of the pressure and flow sensors were transmitted to a notebook by Bluetooth. Figure 1a shows the cuff pressure signal. After the cuff pressure deflated to 55 mm Hg and stayed there for about 8 s, the pulse signal was extracted. The oscillometric waveform and pulse signals were shown in Figure 1b. The blood pressures, the systolic and diastolic pressures, were measured by oscillometry within the deflating duration. SV was measured within the holding duration using Romano’s method.

### 2.2. Compliance of Brachial Artery

The pumped airflow and cuff pressure were simultaneously recorded during the inflation period, with airflow converted into the volume by integration. The cuff volume and pressure were used to construct the cuff model with an exponential function [4]:

(1) $$V_{cuff}=V_{cuff\_0}+a\left(1-e^{-bP_{cuff}}\right)$$

where *V_cuff_* is the volume of air pumped to the cuff, *V_cuff__*_0_ the initial air volume in the cuff, and *P_cuff_* is the air pressure inside the cuff during the inflation period. Therefore, the slope of the cuff model, *C_cuff_*, is defined as

(2) $$C_{cuff}\left(P_{cuff}\right)=\frac{dV_{cuff}}{dP_{cuff}}$$

According to Equation (2), during the deflation period, the pulse signal in the cuff pressure resulted from the change in the arterial volume (*V_pulse_*). Therefore, when the cuff pressure was deflated to 55 mm Hg and stayed there, the pulse change of the arterial volume (Δ*V_pulse_*) embedded in the cuff volume can be calculated from *C_cuff_* and amplitude of the pulse signal (*Amp_puls_*) in the cuff pressure:

(3) $$\Delta V_{pulse}\left(P_{cuff}\right)=C_{cuff}\left(P_{cuff}\right)Amp_{pulse}\left(P_{cuff}\right)$$

Thus, the loaded compliance of brachial artery (*C_artery_*) under the different cuff pressures is defined as the following equation [12]:

(4) $$C_{artery}\left(P_{cuff}\right)=\frac{\Delta V_{pulse}\left(P_{cuff}\right)}{\Delta P_{pulse}}$$

where Δ*P_pulse_* is the brachial artery pulse pressure. According to this formula, *C_artery_* could be considered to be a function of *P_cuff_*, and be used to elucidate the properties of the arterial compliance.

### 2.3. Resistance of Peripheral Artery

In the two-element Windkessel model, the whole arterial tree is modeled as an elastic chamber with a peripheral arterial compliance, *C*, and a peripheral resistance, *R*. The governing equation of the two-element Windkessel is

(5) $$C\frac{dP\left(t\right)}{dt}+\frac{P\left(t\right)}{R}=F\left(t\right)$$

where *F* denotes the arterial blood flow and *P* is the arterial blood pressure. The peripheral arterial compliance was replaced with *C_artery_* in this study. Therefore, during diastole, there is no blood inflow from the heart (*F*(*t*) = 0), and hence the right-hand side of Equation (5) vanishes and a direct integration yields

(6) $$P\left(t\right)=P\left(t_{1}\right)e^{-\left(t-t_{1}\right)/R\cdot C_{artery}},$$

where *P*(*t*_1_) is the beginning blood pressure in the diastolic phase. Equation (6) expresses a monoexponential decay and can be fitted to any portion of the diastolic waveform to yield the time constant, *τ*:

(7) $$\tau =R\cdot C_{artery}$$

### 2.4. Stroke Volume Measurement

According to the pulse contour method combined with the Windkessel model, if the total peripheral resistance and arterial compliance are known, SV can be estimated. In this study, we used the method proposed by Romano et al. to estimate the SV [13],

(8) $$\mathrm{SV}=\frac{A}{dP\cdot R}$$

where *A* is the integrative area of the pressure waveform under the systolic duration.

(9) $$dP=\frac{P_{sys}-P_{dia}}{t_{1}}+\frac{P_{3}}{T-t_{3}}-\frac{P_{2}}{T-t_{2}}$$

**Figure 2:**
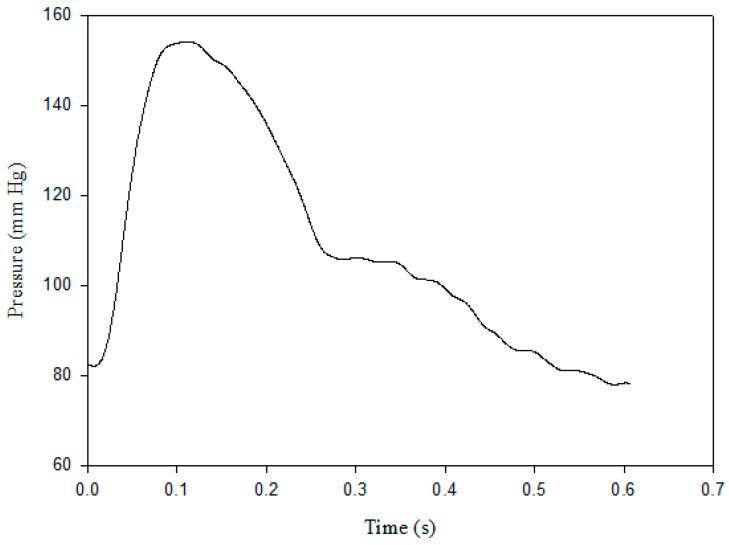
The definitions of pressure and time symbols on the blood pressure contour.

In Figure 2, *P_sys_* is the pressure occurring at the time (*t*_1_) of the maximum value of the pressure waveform, *P_dia_* is the minimum value of the pressure waveform, *P*_2_ is the pressure occurring at the time (*t*_2_) of the zero value of the second derivative of the pressure waveform between *P_sys_* and *P*_3_, and *P*_3_ is the pressure after the main peak of the pressure waveform occurring at the time (*t*_3_) of the dicrotic notch which was the zero value of the first derivative of the pressure waveform. *T* is the time of the cardiac cycle.

When cuff pressure deflated to 55 mm Hg and stayed here for 8 s, we chose the pulses with the complete contours within this time. Then, the diastolic waveform of the pulse contour was fitted by Equation (6) for each beat. The three beats with the highest correlation coefficients were selected to calculate SV with the averages of their SV parameters, *A*, *R*, and *dP*.

### 2.5. Experimental Protocol

Twenty-four healthy adults (12 men and 12 women) with ages of 40.9 ± 9.3 years (mean ± SD; range: 23–55 years) participated in this study, their weights were between 46 kg and 83 kg (65.9 ± 10.6 kg), their heights were between 152 cm and 186 cm (167.3 ± 9.2 cm), with no cardiac diseases or injured limbs. This experiment was approved by the Research Ethics Committee of China Medical University & Hospital (No. CMUH106-RREC1-137).

An ICG instrument was used to measure the stroke volume during the PLR test (Medis^®^ CS 1000, Medizinische Messtechnik GmbH, Ilmenau, Germany). This instrument has a function that can do the SV measurement for a PLR test. In the PLR program, the SV was measured two minutes before the PLR, and the Medis^®^ CS 1000 established their stable SV at this time and used that as the baseline, *SV_base_*. SV was measured again two minutes later while the subjects were lifting their legs. The maximum SV value was detected as the reactive sample, *SV_sample_*. The changed ratios and differences in SV were defined as

Changed ratio of SV = *SV_sample_*/*SV_base_* (10)

Changed difference of SV = *SV_sample_* − *SV_base_* (11)

**Figure 3:**
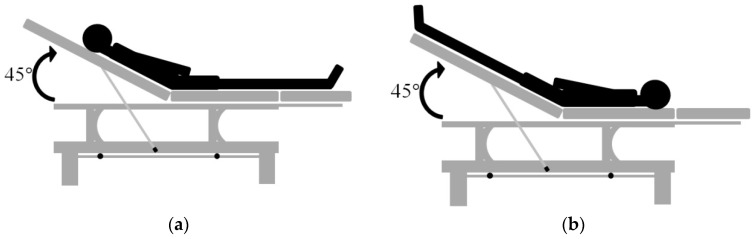
The schematic diagram of passive leg raising PLR test: (**a**) The seating posture of subject before PLR test and (**b**) the lying posture of subject under PLR test.

In this study, the top part of a bed was raised. The subjects sat on the bed, and placed their legs horizontally on the bed (Monnet et al., 2008), as shown in Figure 3a. We attached the cuff of blood pressure monitor to their left forearm and while the Medis^®^ CS 1000 was continuously measuring SV under these stable conditions, our device started its measurements. We recorded the time we started and when this measurement was finished, the subjects were requested to quickly change their posture. They then laid on the bed with their legs lifted at a 45° angle, as shown in Figure 3b. Then the Medis^®^ CS 1000 measured their SV again. When the changes in the SV rose, our device started its measurements, and that time was also recorded.

## 3. Results

Table 1 shows the results of all the subjects. The systolic pressures measured by our device were 117.0 ± 17.1 mm Hg and 115.9 ± 16.1 mm Hg before and during the PLR test, respectively. The diastolic pressures were 80 ± 11.7 mm Hg and 74.9 ± 10 mm Hg before and during the PLR test, which show very significant differences (*p* < 0.01). The stroke volumes measured by the pulse contour method were 60.3 ± 22.1 mL and 67.1 ± 22.1 mL before and during the PLR test, respectively, which also show significant differences (*p* < 0.05). The stroke volumes measured by the Medis^®^ CS 1000 are 67.3 ± 13.7 mL and 91.2 ± 11.4 mL before and during the PLR test, respectively, which also have very significant differences (*p* < 0.01). There were four subjects whose changed ratios in the SV, measured by the pulse contour method, were less than one when doing the PLR test. There was one subject whose changed ratios in the SV, measured by the pulse contour method and the Medis^®^ CS 1000, were less than one.

**Figure 4:**
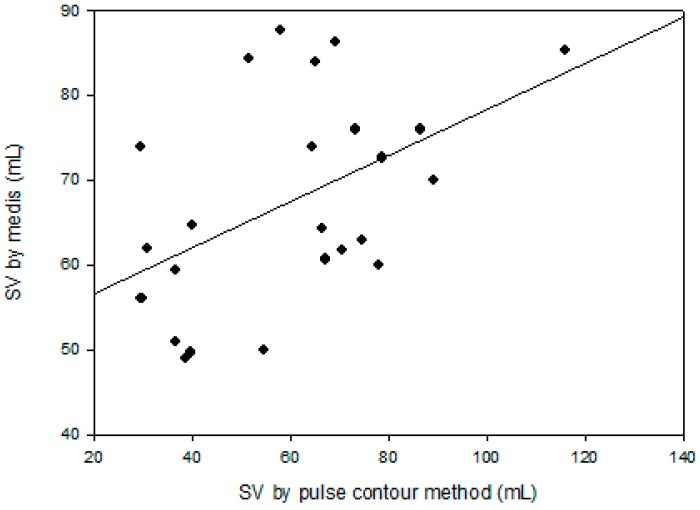
The SV with using the pulse contour method and the Medis, correlation coefficient, *r*^2^ = 0.237.

**Figure 5:**
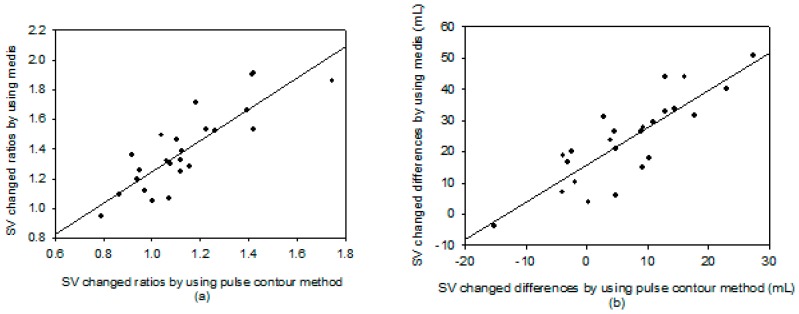
(**a**) The correlation of changed ratios in the SV between the pulse contour method and Medis, *r*^2^ = 0.712. (**b**) The correlation of changed differences in the SV between the pulse contour method and Medis, *r*^2^ = 0.709.

**Figure 6:**
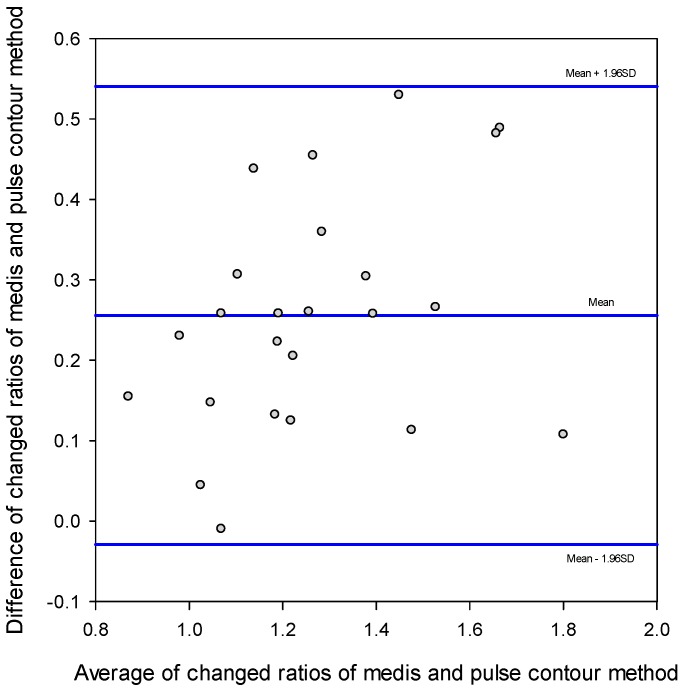
The Bland–Altman plot of the changed ratios of SV measurement using the pulse contour method and the Medis^®^ CS 1000. Mean: 0.256. Standard division (SD): 0.145. Limits of agreement: −0.029 and 0.540.

The correlation coefficient, *r*^2^, between the SV measurements with the pulse contour method and the Medis^®^ CS 1000 was only 0.237, as shown in Figure 4. But, the correlation between the changed ratios of the SV measurement using the pulse contour method and the Medis^®^ CS 1000 was very high. The correlation coefficient, *r*^2^, was 0.712, as shown in Figure 5a. Figure 5b shows the correlation between the changed differences in the SV measurement using the pulse contour method and the Medis^®^ CS 1000. The correlation coefficient, *r*^2^, was 0.709. Figure 6 shows the Bland–Altman graph for the differences and the average of the changed ratios of SV measurement by using the pulse contour method and the Medis^®^ CS 1000. The mean and standard deviation of the difference were 0.256 ± 0.145, the limits of agreement were −0.029 and 0.540. All data points were within the agreement.

## 4. Discussion

Hemodynamic monitoring of SV, CO, and BP are important and useful in many clinical diagnoses and therapies, such as serious heart failure and cardiogenic shock [14,15], early detecting the resuscitation of progressive hemorrhage and hypovolemic shock [16], fluid responsiveness in patients undergoing gastrointestinal surgery [17], and detecting the changes in the aortic pressure due to orthostatic-related syncope [18]. There are now some methods that are acceptable for noninvasive SV measurement such as echocardiography and impedance cardiography. The Transvalvular Doppler echocardiogram measurement was the true gold standard in the clinical measurement of SV [1,19], but it requires an experienced technician. Whole-body impedance cardiography was also limited by some factors, such as restlessness, arrhythmia, or shunts [20]. However, the intended uses of these cardiographs are in the hospital environment and carried out by nurses or doctors. They could not be used in a home environment.

In recent years, several studies based on the pulse contour method attempted to provide reliable, noninvasive, and continuous CO measurements [11,12,13]. But, in the Windkessel model, the SV measurement uses the pulse contour of the central artery, not the peripheral artery. Thus, in this study, the accuracy of the SV measurement was less accurate because we used the pulse contour of the brachial artery. Moreover, the correlation coefficient, *r*^2^, between the SV measurements with the pulse contour method and the Medis^®^ CS 1000 was only 0.237. The invasive method for the SV measurement is a golden standard, like as thermodilution. Therefore, the ICG instruments all used the thermodilution method to verify the accuracy of the absolute SV in clinical trials [6,21]. Thus, the reason for the lower accuracy of the SV measured by the pulse contour method may be due to using the noninvasive measurement method as the reference standard. However, monitoring changes in SV for patients that suffer from heart failure is an important factor in the cardiac care unit [22]. Thus, we assessed the performance of this method for SV measurement with the PLR test. In Table 1, the pulse pressures and the SV taken with our designed device during the PLR test were significantly higher than before the PLR test (41.0 ± 8.8 mm Hg vs. 37.0 ± 6.7 mm Hg, and 67.1 ± 22.1 mL vs. 60.3 ± 22.1 mL). The SV with the Medis^®^ CS 1000 also has a significant difference. These results were the same as the outcomes of Monnet’s study [12]. The correlation coefficient of the changes in the ratio and the difference of SV using the Medis^®^ CS 1000 and pulse contour method were very high, *r*^2^ = 0.712 and *r*^2^ = 0.709, respectively. This results shows that the pulse contour method could be used to measure the changes in the SV.

**Figure 7:**
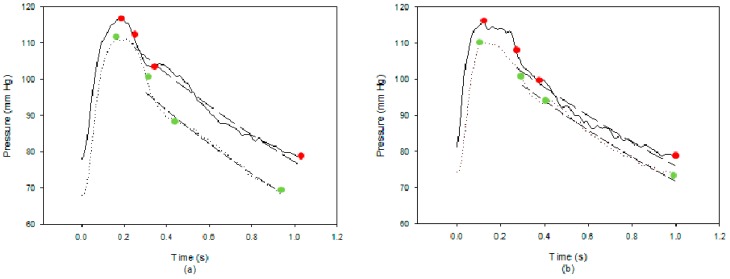
The blood pressure contours and curve fitting results before PLR test (solid line) and during PLR test (dot line) (**a**) for subject 17 and (**b**) for subject 16.

We analyzed the difference between the pulse contour of the brachial artery before the PLR test (solid line) and also during the PLR test (dotted line) for subject 17. Before the PLR test, the subject’s BP was 117/78 mm Hg, SV was 87 mL when using our designed device, and the SV was 76 mL using the Medis^®^ CS 1000. During the PLR test, the subject’s BP was 111/68 mm Hg and the SV was 98 mL when using our designed device, the SV was 105 mL using the Medis^®^ CS 1000, as shown in Figure 7a. The changed difference in the SV for this subject using the pulse contour method was 11 mL, and the difference was 29 mL using the Medis^®^ CS 1000. The pressure symbols before the PLR test were red and green during the PLR test. The diastolic duration of the pulse contour had the best evident exponential decay. The long dashed line describes the curve fitting result for the pulse contour before the PLR test. The correlation coefficient was *r*^2^ = 0.977. The short dashed line describes the curve fitting result for the pulse contour during the PLR test. The correlation coefficient was *r*^2^ = 0.987. We found that the dicrotic notch of pulse contour occurring time, *t_3_*, during the PLR test was longer than the time before the PLR test. The morphologies of the two pulse contours were also different.

Figure 7b shows the pulse contours of the brachial artery before the PLR test (solid line) and during the PLR test (dotted line) for subject 16 whose BP was 116/78 mm Hg, SV was 79 mL using our designed device, and SV was 73 mL using the Medis^®^ CS 1000 before the PLR test, and BP were 110/73 mm Hg and SV was 75 mL using our designed device, and SV was 91 mL using the Medis^®^ CS 1000 during the PLR test. In the diastolic duration, the long dashed line describes the curve fitting result for the pulse contour before the PLR test. The correlation coefficient was *r*^2^ = 0.962. The short dashed line describes the curve fitting result for the pulse contour during the PLR test. The correlation coefficient was *r*^2^ = 0.985. The changed difference in the SV for this subject using the pulse contour method was −4 mL, and the difference using the Medis^®^ CS 1000 was 18 mL. We found that the morphologies of the two pulse contours were almost the same. This represents that the sensitivity of measuring the changing of the SV could be lowered using the pulse contour method when the changed difference of the SV is smaller. In the Bland–Altman plot (Figure 6), the mean of difference was 0.256, which also explained the phenomenon.

## 5. Conclusions

The measuring procedure of the pulse contour method device was very easy, similar to the commercial blood pressure monitor. Although its accuracy for SV measurement was lower, it could measure the changes in the SV. Because this method can be implemented in the blood pressure monitor, people will not only understand the changes in their blood pressure, but also understand the changes in their SV every day. Thus, the pulse contour method could be beneficial for use in home care or a care center for the elderly.

## Figures and Tables

**Table 1:** The results of all the subjects.

	i-BP 130		Medis	
	Before	Under	Before	Under
Sys (mm Hg)	117.0 ± 17.1	115.9 ± 16.1		
Dia (mm Hg)	80.0 ± 11.7	74.9 ± 10.0 **		
PP (mm Hg)	37.0 ± 6.7	41.0 ± 8.8 *		
SV (mL)	60.3 ± 22.1	67.1 ± 22.1 *	67.5 ± 12.4	91.2 ± 11.4 *

*: *p* < 0.005, **: *p* < 0.001. Sys: systolic pressure, Dia: diastolic pressure, PP: pulse pressure, SV: stroke volume, mean ± standard division.
